# Rate of Total Hip Replacement after Legg Calve Perthes Disease in a Canadian Province

**DOI:** 10.3390/pediatric15040053

**Published:** 2023-10-07

**Authors:** Jonathan Tan, Anirudh Sharma, Rohit Bansal, Qier Tan, Heather J. Prior, Sheila McRae, James R. McCammon

**Affiliations:** 1 Division of Orthopaedic Surgery, University of Manitoba, Winnipeg, MB R3A 1R9, Canada; 2 School of Medicine, University of California San Francisco, Fresno, CA 93701, USA; 3 Manitoba Centre for Health Policy, University of Manitoba, Winnipeg, MB R3E 3P5, Canada

**Keywords:** outcomes, pediatric orthopedics, perthes disease, total hip arthroplasty

## Abstract

Legg Calve Perthes disease is a pediatric hip condition that leads to early hip degeneration. The efficacy of operative and nonoperative treatment is not well defined in the literature. Using the rate of total hip arthroplasty as a surrogate measure for symptomatic hip degeneration, the rate of total hip arthroplasty was compared in Legg Calve Perthes disease patients with and without previous surgical intervention in the province of Manitoba, Canada. A retrospective review was conducted using de-identified, individual-level administrative records of health services for the entire population of Manitoba. Codes for Legg Calve Perthes disease, femoral osteotomies, pelvic osteotomies, adductor tenotomies, and total hip arthroplasty were searched from 1984 to 2018. The rate of total hip arthroplasty in patients with Legg Calve Perthes disease was determined for two groups: (1) patients with earlier surgical intervention and (2) patients with no previous surgical intervention. Of the 202 patients included in the study, 180 had no prior surgery and 22 had prior surgery. The rate of total hip arthroplasty between the previous operative and nonoperative groups was found to be 32% and 40%, respectively (*p* = 0.458). There was no significant difference in rates of total hip arthroplasty in the operative and nonoperative groups. Further prospective studies are required to elucidate the differences in outcomes between operative and nonoperative treatment groups in patients with Legg Calve Perthes disease.

## 1. Introduction

**Figure 1:**
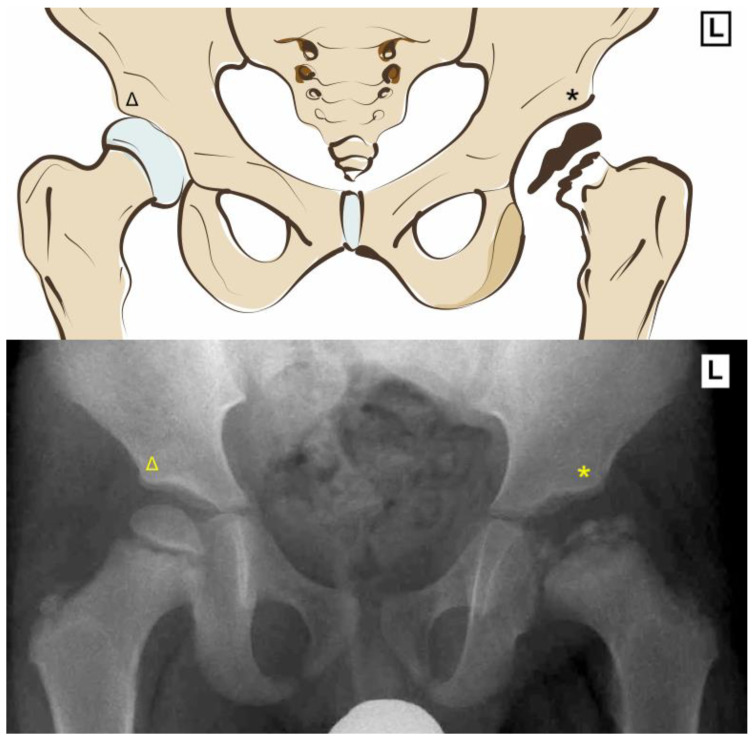
Radiograph and diagram showing difference between the rounded normal ball-and-socket right hip joint (Δ) vs. the asymmetric, fragmented and necrosed left femoral head (*) in a patient with Legg Calve Perthes disease.

Legg Calve Perthes disease involves idiopathic osteonecrosis of the femoral epiphysis with subsequent revascularization. The loss of blood supply leaves the head vulnerable to deformation with the risk of development of an aspherical femoral head and incongruent joint (Figure 1). Stulberg found that an aspherical femoral head (class III) has a 58% chance of developing radiographic signs of osteoarthritis 40 years from presentation, whereas a spherical head (class I–II) has a 0–16% chance of radiographic osteoarthritis at 40 years [1].

The goal of interventions for Legg Calve Perthes disease is to increase the contact area between articular surfaces through containment, thereby reducing edge loading on the femoral head. The optimal treatment strategy is difficult to define, as there is literature that supports both operative and nonoperative treatment [2], depending on age and radiographic staging [3]. Biologic interventions [3] to supplement current surgical interventions appear promising, but these are still in development. Operative treatment consists of femoral, pelvic or combined osteotomies. However, if these containment measures fail or the disease progresses, total hip arthroplasty is an effective option. Recent trends have shown an increase in the rates of all-cause total hip arthroplasties around the globe: USA, +12.87%; Australia, +7.77%; United Kingdom, +6.95%; Germany, +6.07%; Spain, +6.73%; and Romania, 8.19% [4]. This increase can be attributed in part to expanded indications, better surgical techniques, implant longevity and improved outcomes.

The rate of total hip arthroplasty in patients with Legg Calve Perthes disease has been found to correlate with hip function scores [5]. In the literature, 0 to 43% of nonoperatively treated patients with Legg Calve Perthes disease eventually have a total hip arthroplasty [6]. Proximal femoral varus osteotomies are reported to have a 17% rate of conversion to total hip arthroplasty at a mean 42.5 year follow-up [7]. The average age at the time of total hip arthroplasty was 37.8 years [8]. There is limited research about whether patients with prior containment surgery do better compared to conservative management. Specifically, the rates of total hip arthroplasty have not been well studied. Although systematic and narrative reviews suggest comparable results [9,10], these studies suffer from high heterogeneity, small sample sizes, and variable follow-up.

Therefore, the primary objective of this study was to compare the rate of total hip arthroplasty in Legg Calve Perthes disease patients who underwent previous surgery to that of those who had nonoperative treatment. The rate of total hip arthroplasty will act as a surrogate measure for symptomatic hip degeneration and may provide insight into the long-term impact of surgical intervention. Our hypothesis was that prior surgical intervention may improve hip function, thereby decreasing the overall rate of total hip arthroplasty, assuming appropriate surgical indications and techniques were followed.

## 2. Materials and Methods

### 2.1. Study Design

**Figure 2:**
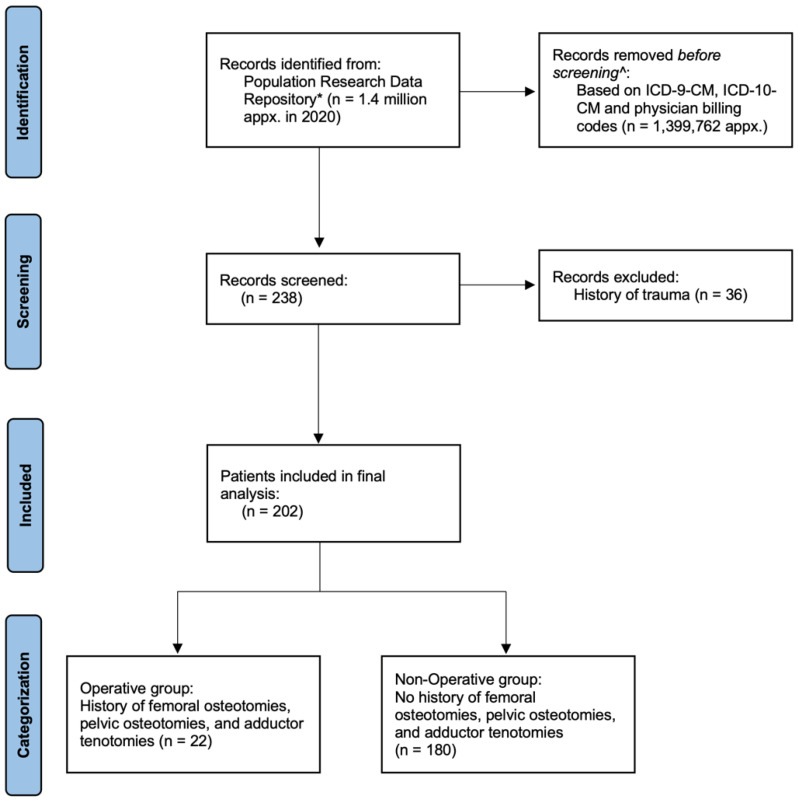
Flow diagram showing methodology, inclusion, and exclusion criteria. * The repository contains de-identified linkable information on health care delivery and utilization for the entire population in the province of Manitoba, Canada. ^ See Appendix A for details.

A retrospective review of de-identified data from the Population Research Data Repository at the Manitoba Centre for Health Policy was performed for the entire population of Manitoba (approximately 1.4 million in 2020). The repository contains de-identified linkable information on health care delivery and utilization for the entire population in the province of Manitoba, Canada, and includes a comprehensive collection of administrative, survey, registry, and clinical datasets maintained by the provincial ministry of health and other provincial ministries. The Manitoba Health Insurance Registry covers care related to physicians, hospitals, personal care homes, home care, and pharmaceutical prescriptions. This analysis used data from the Manitoba Health Insurance Registry, hospital discharge abstracts, and medical services to collect demographics as well as diagnostic and surgical billing codes. Further information regarding the Manitoba Centre for Health Policy database can be found in the Appendix Section. Figure 2’s flow diagram exhibits the methodology in further detail.

### 2.2. Data Collection

Diagnosis codes from hospital abstract data (ICD-9-CM code 732.1; ICD-10-CM code M91.0, M91.1, M91.2, M91.3) were used to identify Legg Calve Perthes disease patients. Intervention codes for hospital abstracts (CCI codes 1.SQ.80 and 1.VC.80) and physician billing codes (20532, 20539, 21337, 21338, and 21339) were used to identify their surgical treatments (osteotomy) from 1 January 1984 to 31 December 2018.

The ICD-9-CM diagnosis code 732.1 was specified as “juvenile osteochondrosis of hip and pelvis”. Operative treatment included femoral osteotomies, pelvic osteotomies, and adductor tenotomies. Nonoperative treatment was defined as the absence of surgical intervention.

A total of 238 patients with a diagnosis of Legg Calve Perthes disease were identified. A total of 36 patients who received total hip arthroplasty secondary to trauma were excluded. Trauma was identified through ICD-9-CM diagnosis codes E800-E848, E881-E884, E908-E909, and E916-E928 as well as ICD-10-CA diagnosis codes V01-V99, W11-W17, W20-W49, W85-W99, X10-X19, X34-X39 and X50-X59.

Patient demographics, including age, gender, geography (urban vs. rural) and income quintile, were identified. Income quintile was determined by the residential postal code. This was then linked to the Canada Census data for the mean household income of the corresponding neighborhood. Urban centers included Winnipeg and Brandon, whereas rural areas included the surrounding areas and the rest of Manitoba. Study approval from the local research ethics board was obtained. Individual consent was not required for the study.

### 2.3. Statistical Analysis of Data

Descriptive statistics were generated for demographic data. Age was analyzed as a continuous variable, whereas gender, geography, and income quintile were analyzed as categorical variables. Frequency data were compared with chi-squared tests, and a significance level was set at *p* < 0.05. Data analysis was carried out using SAS version 9.4.

## 3. Results

**Figure 3:**
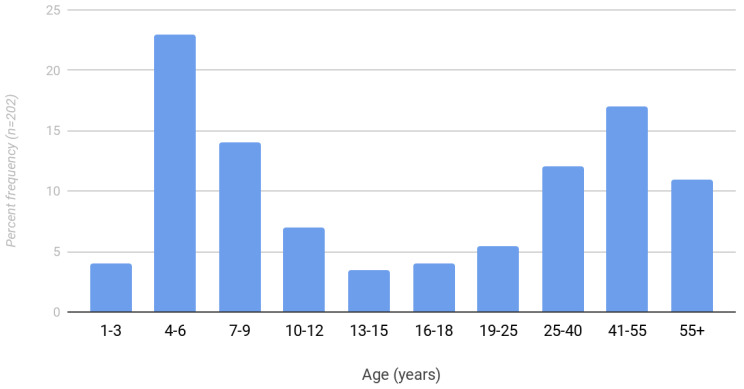
Frequency distribution of age at diagnosis for Legg Calve Perthes disease patients between 1984–2018.

**Figure 4:**
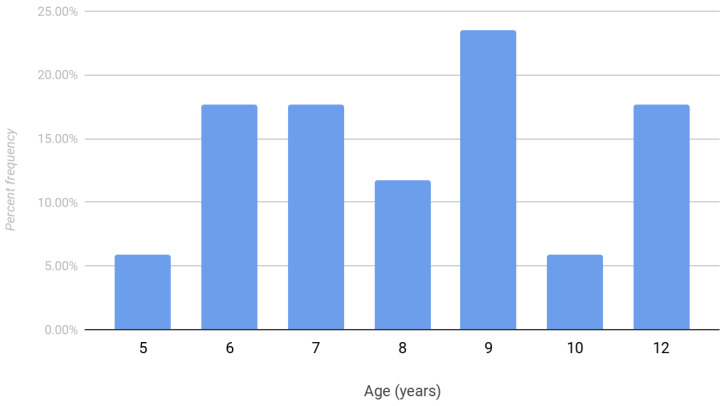
Frequency distribution of age of Legg Calve Perthes disease surgical intervention.

Demographic information is presented in Table 1. The rate of total hip arthroplasty between the operative and nonoperative groups was found to be 32% and 40%, respectively (*p* = 0.458; Table 2). The mean age of total hip arthroplasty in the operative group was 33.9 years (standard deviation 9.4) compared to 46.3 years (standard deviation 13.5) in the nonoperative group. There was also a bimodal distribution of pediatric and adult diagnoses of Legg Calve Perthes disease (Figure 3). A total of 59% of patients were diagnosed after age nine. The frequency of surgical intervention in patients with Legg Calve Perthes disease is shown in Figure 4. The mean age at the time of prior surgical intervention was 8.4 years.

## 4. Discussion

### 4.1. Primary Finding

The present study found no significant differences in the rates of total hip arthroplasty between Legg Calve Perthes disease patients who underwent prior surgery and those who had nonoperative treatment. This shows that careful indications are needed to determine the best surgical candidates for early surgical intervention, such as a proximal femoral varus osteotomy.

Herring reported similar outcomes between operative and nonoperative treatment groups in patients under age 6 [3] and between the ages of 6 and 8 years [11]. Nevertheless, for ages > 8 years, Herring found improved rates of radiographic outcomes with proximal femoral varus osteotomy (62% resulting in Stulberg I–II hips) compared to the no treatment group (25% resulting in Stulberg I–II hips). However, these results were not statistically significant, possibly due to the study being underpowered. When analyzed with the lateral pillar classification, a beneficial effect was found with surgery compared to nonoperative treatment for lateral pillar B and B/C border groups [3]. A recent systematic review by Caldaci et al. reported that the main prognostic factors for surgical outcome were the age at onset and the degree of initial disease severity [12]. Surgical treatment in patients older than 6 years showed excellent results in Herring B and B/C hips and poor results in Herring C hips, with a slight advantage for patients between 6 and 8 years old.

When not accounting for age or lateral pillar classification, Herring’s study found a statistically significant improvement (*p* = 0.02) with surgical intervention compared to nonoperative treatment. A total of 46% of patients treated nonoperatively had a spherical head at maturity. Conversely, operative treatments resulted in 61% patients with a spherical head at maturity. The sphericity of the femoral head has been found to correlate with the rate of total hip arthroplasty in Legg Calve Perthes disease [13]. At a mean 47 year follow-up, Froberg found that only 9% of patients with spherical heads resulted in total hip arthroplasty compared to 27% with non-spherical heads (*p* = 0.04) [13].

A 2012 meta-analysis [14] of 23 studies consisting of 1232 patients found that children under the age of 6 had good outcomes (*p* = 0.828), whereas children older than 6 were nearly twice as likely to achieve a spherical head with surgical intervention (*p* < 0.0001).

### 4.2. Secondary Findings

There were notable secondary findings. First, the operative group underwent conversion to total hip arthroplasty at an earlier age than the nonoperative group. Second, the rates of total hip arthroplasty were found to be significantly higher in both operative and nonoperative groups compared to the general population. Finally, there was a high rate of diagnoses above age 9.

#### 4.2.1. Age at Time of Total Hip Arthroplasty

In the literature, it appears that the age of total hip arthroplasty is correlated to the degree of joint incongruity as measured by the Stulberg classification, which tends to be worse with nonoperative treatment. However, in this study, we found that the operative group underwent total hip arthroplasty at a younger age than the nonoperative group (33.9 years, standard deviation 9.4 compared to 46.3 years, standard deviation 13.5). This trend towards a younger age could be due to the initial severity of presentation, requiring an earlier surgery, such as a proximal femoral varus osteotomy. It is also relevant in terms of planning revision surgeries. A patient receiving a total hip arthroplasty in their thirties may require at least one revision, with the index procedure potentially requiring specialized implants to accommodate a prior proximal femoral varus osteotomy.

#### 4.2.2. Rate of Total Hip Arthroplasty

In both groups, 32–40% of patients over 34 years eventually received a total hip arthroplasty, which is higher than what is seen in most studies. In 2010, a review by Kim [3] showed that most patients with a mean follow-up <40 years remained active and lacked symptoms, despite a femoral head deformity. This shows that the rate of conversion to total hip arthroplasty may be underreported in the literature and is important to discuss with families when formulating a treatment plan.

Legg Calve Perthes disease patients are also at a higher risk of requiring a total hip arthroplasty at an earlier age compared to the general population. In the US, the rate of total hip arthroplasty is approximately 0.9% in the age group 50–59 in the general population [15]. In Manitoba, the rate of total hip arthroplasty in the general population aged between 40 to 49 years [16] was 0.039% from 1999–2017 as per the Population Research Data Repository at the Manitoba Centre for Health Policy. This study showed that both operative and nonoperative treatments resulted in a more than thirty-fold increased risk of total hip arthroplasty. Given the high risk of total hip arthroplasty, it is essential to carefully select patients for surgical intervention, as proximal femoral osteotomies can make future total hip arthroplasty technically challenging. Furthermore, alternative surgical interventions, such as percutaneous transepiphyseal drilling of the femoral head combined with closure of the greater trochanter apophysis, can be an attractive option during early stages of fragmentation (Stulberg type 1–2) as this does not compromise any potential future treatment options [17]. Similarly, a recent study by Moličnik et al. found that biomechanical changes caused by deformed hip articulation play a critical role in the development of secondary hip arthritis in patients with Legg Calve Perthes disease [18]. The authors found a significant association between the radiological hip containment parameter (small center-edge angle) and an unfavorable distribution of stresses across the hip articulation. Thus, in addition to clinical measures, a small center-edge angle on imaging could be considered as an additional measure to help guide decision-making regarding containment surgery or to predict post-containment outcomes. This merits further investigation.

#### 4.2.3. Age at Time of Diagnosis

Finally, we found that the majority of patients in this study presented late (59% over age 9, 52% over age 12). Older age at the time of diagnosis could in part be attributed to the non-specificity of diagnosis code 732.X in outpatient claims data resulting in a failure to include these patients in our analysis. Other possible reasons for late presentation could be the late onset of symptoms, milder symptoms, or a delay in an appropriate diagnosis.

Older age at presentation can be associated with advanced disease, especially if diagnosed late. These patients may be better suited for nonoperative treatment, given the high rates of total hip arthroplasty. In contrast, Arkader reported that there was a trend towards improved radiographic appearances with the late onset group over age 8 [19]. While it is possible that a proximal femoral varus osteotomy may improve radiographic appearance, our study demonstrates that long-term outcomes in this group may not necessarily correspond to an early improved radiographic appearance.

Furthermore, the large number of late presenters may be due to the fact that 50% of cases were referred from rural settings and that 49% patient belonged to lower income quintiles. These patients may have had limited access to timely primary or orthopedic care. The role of primary care education is essential in avoiding delay in diagnosis, as a timely intervention can improve outcomes, especially when the femoral head is vulnerable during the fragmentation phase. 

### 4.3. Limitations

There are important limitations in this study. Firstly, in terms of the study design, there was a relatively small number of patients who underwent previous surgical treatment for Legg Calve Perthes disease (22 out of 202 patients). Secondly, since this was a database study with de-identified patient information, there were no radiographic images. As a result, patients could not be analyzed according to the lateral pillar, Waldenstrom, or Stulberg classifications, which may have resulted in differences in outcomes among particular subgroups. Thirdly, we could not differentiate Legg Calve Perthes disease from other osteochondropathies within the outpatient medical claims data, since ICD-9-CM diagnosis codes were limited to the first three digits, which lacked diagnostic specificity. Alternatively, we relied solely on hospital abstracts with the diagnosis codes 732.1 or M91.x. This could have led to an artificial late disease presentation, since many patients could have presented sooner to a general physician or specialist at an outpatient setting and were not included in our analysis. Furthermore, a large proportion of patients over age 12 presenting to hospitals would result in many children being poor candidates for containment therapy, which would reduce the efficacy of surgical interventions.

## 5. Conclusions

In conclusion, patients with Legg Calve Perthes disease have a relatively higher rate (32–40%) of total hip arthroplasty compared to the general population, with no difference in the rate of total hip arthroplasty (*p* = 0.458) among operative and nonoperative treatment groups. This high rate of total hip arthroplasty compared to the general population may have resulted from 59% of patients presenting after age 9, which could have resulted in a more severe pathology with less remodeling time.

While the current literature supports operative intervention to improve the chances of developing a spherical head, our study did not show any difference in the rates of total hip arthroplasty in patients with prior operative treatment compared to nonoperative management. This emphasizes the importance of early diagnosis and careful patient selection for surgical treatment. Analyzing our results along with the pre-existing literature, we conclude that early surgery, as early as age 6, is ideal, especially in the presence of lateral subluxation and Herring B/C hips. We also recommend an informed discussion with families with regards to the high rates of total hip arthroplasty in patients who do not fit this profile, particularly older patients.

In addition to refining surgical and biological techniques, improved education for primary care providers may help them identify Legg Calve Perthes disease in its earlier stages and expedite referral to a pediatric orthopedic surgeon, which may lead to more favorable outcomes. Further prospective studies are required to better elucidate the differences in outcomes between operative and nonoperative treatment groups in patients with Legg Calve Perthes disease.

## Figures and Tables

**Table 1:** Demographic information of all Legg Calve Perthes disease patients.

Demographics	
N	202
Gender	
Male	75.2%
Female	24.8%
Residence	
Urban	50.0%
Rural	50.0%
Income quintile	
N/A	0.5%
Q1 (lowest)	25.7%
Q2	23.3%
Q3	11.9%
Q4	18.8%
Q5 (highest)	19.8%

**Table 2:** Rate of total hip arthroplasty in Legg Calve Perthes disease patients from 1984 to 2018.

	Rate (n (%))
Overall	79/202 (39%)
With previous surgical intervention	7/22 (32%)
Without previous surgical intervention	72/180 (40%)
*p*-value	0.458

## Data Availability

The results presented in this study are available on reasonable request from the corresponding author. The individual level data are not available due to privacy and confidentiality rules required by the Manitoba Ministry of Health.

## Appendix A

The following are descriptions of the databases [16] used:

Hospital abstracts: Health data maintained by Manitoba Health consisting of hospital forms/computerized records containing summaries of demographic and clinical information (e.g., gender, postal code, diagnoses, and procedure codes) completed at the point of discharge from the hospital. Several hundred thousand abstracts per year are submitted for all separations from acute and chronic care facilities in Manitoba and for all Manitobans admitted to out-of-province facilities. The Hospital Abstracts Data includes records of both Manitoba residents and non-Manitoba residents hospitalized in Manitoba facilities and information about inpatient and day surgery services.

Medical claims: Health data maintained by Manitoba Health consisting of claims for physician visits in offices, hospitals, and outpatient departments; fee-for-service components for tests such as lab and X-ray procedures performed in offices and hospitals; payments for on-call agreements (e.g., anesthetists) that are not attributed to individual patients; as well as information about physician specialties. These data files contain records for both Manitoba and non-Manitoba residents who visit Manitoba providers. Some information is also included for services received by Manitoba residents from providers in other provinces. In Manitoba, fee-for-service providers must submit claims to Manitoba Health for reimbursement; a small proportion of salaried physicians also submit evaluation claims (shadow billing).

Manitoba Health Insurance Registry: A longitudinal population-based registry maintained by Manitoba Health of all individuals who have been registered with Manitoba Health at some point since 1970. The registry includes individual-level demographics, family composition information, residential postal codes, and data fields for registration, birth, entry into province, and migration in/out of province. It provides the needed follow-up information to track residents for longitudinal and intergenerational analyses. Individuals who are insured federally, such as military personnel and federal inmates, are not included in this dataset. RCMP were previously excluded but began to be included as of 1 April 2013. Manitoba Centre for Health Policy receives “snapshot files” of registry data semi-annually from Manitoba Health; these files are central to the use of the Data Repository.
